# Efficiently sampling the realizations of bounded, irregular degree sequences of bipartite and directed graphs

**DOI:** 10.1371/journal.pone.0201995

**Published:** 2018-08-13

**Authors:** Péter L. Erdős, Tamás Róbert Mezei, István Miklós, Dániel Soltész

**Affiliations:** Alfréd Rényi Institute of Mathematics, Hungarian Academy of Sciences, Budapest, Hungary; Max F Perutz Laboratories GmbH, AUSTRIA

## Abstract

Since 1997 a considerable effort has been spent on the study of the **swap** (switch) Markov chains on graphic degree sequences. All of these results assume some kind of regularity in the corresponding degree sequences. Recently, Greenhill and Sfragara published a breakthrough paper about irregular normal and directed degree sequences for which rapid mixing of the swap Markov chain is proved. In this paper we present two groups of results. An example from the first group is the following theorem: let $\vec{d}$ be a directed degree sequence on *n* vertices. Denote by Δ the maximum value among all in- and out-degrees and denote by $|\vec{E}|$ the number of edges in the realization. Assume furthermore that $\Delta <\frac{1}{\sqrt{2}}\sqrt{|\vec{E}|-4}$. Then the swap Markov chain on the realizations of $\vec{d}$ is rapidly mixing. This result is a slight improvement on one of the results of Greenhill and Sfragara. An example from the second group is the following: let **d** be a bipartite degree sequence on the vertex set *U* ⊎ *V*, and let 0 < *c*_1_ ≤ *c*_2_ < |*U*| and 0 < *d*_1_ ≤ *d*_2_ < |*V*| be integers, where *c*_1_ ≤ **d**(*v*) ≤ *c*_2_: ∀*v* ∈ *V* and *d*_1_ ≤ **d**(*u*) ≤ *d*_2_: ∀*u* ∈ *U*. Furthermore assume that (*c*_2_ − *c*_1_ − 1)(*d*_2_ − *d*_1_ − 1) < max{*c*_1_(|*V*| − *d*_2_), *d*_1_(|*U*| − *c*_2_)}. Then the swap Markov chain on the realizations of **d** is rapidly mixing. A straightforward application of this latter result shows that when a random bipartite or directed graph is generated under the Erdős—Rényi *G*(*n*, *p*) model with mild assumptions on *n* and *p* then the degree sequence of the generated graph has, with high probability, a rapidly mixing swap Markov chain on its realizations.

## Introduction

An important problem in network science is to algorithmically construct typical instances of networks with predefined properties, often expressed as graph measures. In particular, special attention has been devoted to sampling simple graphs (in our paper only graphs without parallel edges and loops are considered) with a given degree sequence. In 1997 Kannan, Tetali, and Vempala ([1]) proposed the use of the so-called switch Markov chain approach, which had already been used in statistics. We call this the **swap Markov chain** approach.

The **swap operation** exchanges two disjoint edges *ac* and *bd* in the realization *G* with *ad* and *bc* if the resulting configuration *G*′ is again a simple graph (we denote this operation by *ac*, *bd* ⇒ *ad*, *bc*). (For details see the next section). It is a well-known fact that the set of all possible realizations of a **graphic** degree sequence is connected under this operation. (See, for example, Petersen [2] or Havel [3] and Hakimi [4]). An analogous result applies for the swap operation defined on bipartite graphs. (See, for example, Gale [5]). Here we have to be careful, as not every edge exchange is eligible: on a bipartite graph we must ensure that vertices *a* and *d* belong to different vertex classes.

In the literature, the name **switch operation** is also used, however, in our approach this latter is an operation on integer matrices slightly generalizing the swap operation. (See the sections on the analysis of the swap sequences).

The situation is more complicated in case of directed degree sequences. In this case for every vertex the number of incoming edges (**in-degree**) and the number of outgoing edges (**out-degree**) is given in the **degree bi-sequence**. Here the *ac*, *bd* ⇒ *ad*, *bc* type exchange preserves the degree bi-sequence if both before and after the swap operation *a* and *b* are tails of the directed edges. However, imagine that our graph $\vec{G}$ is a directed triangle $\vec{C_{3}}$ while $\vec{H}$ is the oppositely directed $\overset{\leftarrow }{C_{3}}$. Both graphs have the same degree bi-sequence **d** = ((1, 1, 1); (1, 1, 1)). It is clear that there is only one way to transform the first one into the second one: if we exchange three edges and three non-edges in $\vec{G}$. We will call this operation a **triple swap** and the previously defined “classical” one as a **double swap**. Kleitman and Wang proved in 1973 ([6]) that any two realizations of a given graphic degree bi-sequence can be transformed into each other using these two operations. The same fact was re-discovered in 2010 (see [7]).

The swap Markov chains corresponding to the most common graph models are irreducible, aperiodic, reversible (obey detailed balance), have symmetric transition matrices, and thus have uniform global stationary distributions.

In their paper [1], Kannan, Tetali and Vempala conjectured that all these Markov chains are rapidly mixing. The first rigorous proof in this topic is due to Cooper, Dyer and Greenhill for regular graphs ([8]). Now, twenty years after the KTV conjecture, we are still far, probably very far, from proving it in its full generality. However, many partial results have been proved; those which play some role in this paper are summarized in the following theorem:

**Theorem 1**. *The swap Markov chain mixes rapidly for the following degree sequences*:

(A) **d** *is a regular directed degree sequence*.
(B) **d** *is a **half-regular** bipartite degree sequence*.
(C) **d** *belongs to an **almost-half-regular** bipartite graph*.
(D) **d** *is an almost-half-regular bipartite degree sequence, where every realization must avoid a fixed (partial) matching*.
(E) **d** *is a directed degree sequence with* $2\leq d_{\max}\leq \frac{1}{4}\sqrt{M}$, *where M is the sum of the in-degrees (or out-degrees), and where the set of all realizations under study is irreducible under the double swap operation*.

There exist similar results on (normal) degree sequences as well, but their proofs are not fully “compatible” with the proof of the above results (except case D). To our knowledge there does not exist a fully developed proof machinery which is applicable for all three cases, that is, for normal, bipartite, and directed degree sequences.

The result (A) was proved by Greenhill ([9]). In the proof it is assumed that the set of all realizations of the regular directed degree bi-sequence is irreducible under the double swap operations. (B) is due to Miklós, Erdős, and Soukup ([10]). Half-regularity means that in one class the degrees are the same (i.e., regular), while in the other class the only restrictions are those imposed by graphicality. (C) is due to Erdős, Miklós, and Toroczkai ([11]). Here almost-half-regular means that for any pair of vertices on one side we have |*d*(*v*_1_) − *d*(*v*_2_)| ≤ 1. (D) was proved by Erdős, Kiss, Miklós, and Soukup ([12]). This model will be introduced in detail and its intrinsic connection with directed graphs will be fully explained in the section starting with Lemma 12. Papers [9] and [12] are using slightly different Markov chains on regular directed degree sequences, therefore (D) does not supersede (A). Finally, (E) was proved recently by Greenhill and Sfragara ([13]). Their result has vastly extended the set of (normal and directed) degree sequences for which the rapid mixing of the Markov chain is known (e.g., power-law density-bounded normal degree sequences with parameter *γ* > 2.5). The papers [14] and [15] fully characterize those degree bi-sequences where the set of all realizations is irreducible under the double swap operation.

In this paper we improve on the result of Greenhill and Sfragara on directed degree sequences, and we prove the bipartite analogue of their degree sequence result for simple graphs. We achieve this by applying our technique described in [12]. In addition, we further extend the set of bipartite and directed degree sequences with rapidly mixing Markov chain processes, using a condition on minimum and maximum degrees.

Let **d** be a bipartite degree sequence on the underlying set *U* ⊎ *V*. (So the underlying set is equal to the disjoint union of the two classes).

**Theorem 2**. *Let* **d** *be a bipartite degree sequence on U and V as classes, let* |*E*| *be half of the sum of the degrees, and let* Δ = max **d**. *If*

$$\begin{array}{c} 2\leq \Delta \leq \frac{1}{\sqrt{2}}\sqrt{\left|E\right|}, \end{array}$$ (1)

*then the swap Markov chain on the realizations of* **d** *is rapidly mixing*.

The following result describes another wide range of bipartite degree sequences with rapidly mixing swap Markov chain.

**Theorem 3**. *Let* 0 < *c*_1_ ≤ *c*_2_ < |*U*| = *n and* 0 < *d*_1_ ≤ *d*_2_ < |*V*| = *m be integer parameters and assume that* **d** *satisfies the following properties*:

$$\begin{array}{cc} c_{1}\leq d\left(v\right)\leq c_{2}, & \;\forall v\in V\\ d_{1}\leq d\left(u\right)\leq d_{2}, & \;\forall u\in U. \end{array}$$ (2)

*Furthermore, assume that*

$$\begin{array}{c} \left(c_{2}-c_{1}-1\right)\cdot \left(d_{2}-d_{1}-1\right)\leq \max\{c_{1}\left(m-d_{2}\right),d_{1}\left(n-c_{2}\right)\} \end{array}$$ (3)

*holds. Then the swap Markov chain on the realizations of* **d** *is rapidly mixing*.

We conjecture that a very similar result should apply to the case of normal degree sequences.

Our next two results are about directed degree sequences $\vec{d}$ on the *n* element vertex set *X*. The first one improves the constant $\frac{1}{4}$ in the result of Greenhill and Sfragara to $\frac{1}{\sqrt{2}}$. Moreover, because both double and triple swaps are allowed, the irreducibility condition can be omitted from the theorem.

**Theorem 4**. *Let* $\vec{d}$ *be a directed degree sequence on the n element V as its vertex set. Let* $|\vec{E}|$ *be half of the sum of the degrees, and let* Δ = max{max *d*_*out*_, max *d*_*in*_}. *If*

$$\begin{array}{c} \Delta <\frac{1}{\sqrt{2}}\sqrt{|\vec{E}|-4}, \end{array}$$ (4)

*then the swap Markov chain on the realizations of* $\vec{d}$ *is rapidly mixing*.

Lastly, we show that conditions similar to that of Theorem 3 guarantee rapid mixing of the swap Markov chains on a wide class of directed degree sequences.

**Theorem 5**. *Let* 0 < *c*_1_ ≤ *c*_2_ < *n and* 0 < *d*_1_ ≤ *d*_2_ < *n be integer parameters and assume that graphic degree bi-sequence* $\vec{d}$ *satisfies the following properties*:

$$\begin{array}{cc} c_{1}\leq d_{out}\left(v\right)\leq c_{2}, & \;\forall x\in X,\\ d_{1}\leq d_{in}\left(v\right)\leq d_{2}, & \;\forall x\in X. \end{array}$$ (5)

*Furthermore, assume that*

$$\begin{array}{c} \left(c_{2}-c_{1}\right)\cdot \left(d_{2}-d_{1}\right)\leq 2+\max\{c_{1}\left(n-d_{2}-1\right)+d_{1}+c_{2},\;\;d_{1}\left(n-c_{2}-1\right)+c_{1}+d_{2}\}-n \end{array}$$ (6)

*holds. Then the swap Markov chain, using double and triple swap operations, is rapidly mixing on the realizations of* $\vec{d}$.

The proofs of our results strongly support Greenhill’s observation about the existing arguments ([16]): “In each known case, regularity (or half-regularity) was only required for one lemma, which we will call the critical lemma. This is a counting lemma which is used to bound the maximum load of the flow (see [8, Lemma 4], [9, Lemma 5.6], [10, Lemma 6.15])”—and some newer examples—([16, Section 3], [12, Lemma 18], [13, Lemma 2.5 and Lemma 3.6]). The main task is to prove the critical lemmas (Lemma 11 and 12) for our new conditions (2–6). To that end, we first list the fundamental details from [12].

We would like to mention that after submitting a preprint of an earlier version of this paper on arXiv, Amanatidis and Kleer have contacted us, claiming to have proved that the degree sequences studied here are also “strongly stable” (see [17]).

The next 3 sections lay down the foundations of the swap Markov chain on bipartite and directed degree sequences. These have been originally described in our own paper [12]. For the sake of readability and convenience, we recall in more detail some results from paper [12], as they are crucial in understanding the presented approach.

## Definitions and useful facts

In this section, we recall some well-known definitions and results, furthermore we define our swap Markov chains for the bipartite degree sequences and for the directed degree sequences.

Let *G* be a simple bipartite graph on *U* ⊎ *V*, where *U* = {*u*_1_, …, *u*_*n*_} and *V* = {*v*_1_, …, *v*_*m*_}, and let its **bipartite degree sequence** be

$$\begin{array}{c} d\left(G\right)=(d(U),d(V))=((d\left(u_{1}\right),\ldots ,d\left(u_{n}\right)),(d\left(v_{1}\right),\ldots ,d\left(v_{m}\right))). \end{array}$$ (7)

For a *ac*, *bd* ⇒ *ad*, *bc* swap operation to be valid it is not enough that *ac*, *bd* ∈ *E*(*G*) and *ad*, *bc* ∉ *E*(*G*), we also need that *ad* can be an edge in some realization. In other words, we need that *a* and *d* are in different vertex classes. We will use the name **chord** for any vertex pair *u*, *v* where *uv* can be an edge in a realization, even if we do not know or do not care whether it is an edge or a non-edge in the current realization. We can reformulate the definition of the swap operation: it can be done if *ac*, *bd* ∈ *E*(*G*) and *ad*, *bc* are chords.

Now denote by G the set of all possible realizations of the graphic bipartite degree sequence (**d**(*U*), **d**(*V*)). Consider two different realizations, *G* and *H*, of this bipartite degree sequence. As we already mentioned in the introduction, it is a well-known fact that the first realization can be transformed into the second one (and vice versa) with a sequence of swap operations. Formally, there exists a sequence of realizations *G* = *G*_0_, …, *G*_*i*−1_, *G*_*i*_ = *H*, such that for each *j* = 0, …, *i* − 1 there exists a swap operation which transforms *G*_*j*_ into *G*_*j*+1_. We denote the swap Markov chain as M=(G,P) where the **transition matrix** *P* is the following:

In any realization with probability $\frac{1}{2}$ we stay in the current state (i.e., the chain is lazy) and with probability $\frac{1}{2}$ we uniformly choose two-two vertices *u*_1_, *u*_2_;*v*_1_, *v*_2_ from classes *U* and *V*, respectively. We perform the swap *u*_1_*v*_1_, *u*_2_*v*_2_ ⇒ *u*_1_*v*_2_, *u*_2_*v*_1_ if *u*_1_*v*_1_, *u*_2_*v*_2_ ∈ *E*(*G*) and the resulting graph *G*′ is simple. Otherwise we do not perform a move. The swap moving from *G* to *G*′ is unique, therefore the **jumping probability** from *G* to *G*′ ≠ *G* is:

Prob(G→G′):=P(G′|G)=12(m2)(n2). (8)

The transition probabilities are time- and edge-independent and are also **symmetric**. The chain is lazy, therefore aperiodic. It is also reversible, and so its globally stable stationary distribution is uniform.

Now we turn our attention to the notions and notation to describe Theorems 4 and 5. Literally these theorems are about directed graphs, however, we will use the machinery developed in the paper [12], turning these statements into theorems about bipartite graphs with some restriction on which edges can be used in the realizations.

Let $\vec{G}$ be a simple directed graph (parallel edges and loops are forbidden, but oppositely directed edges between two vertices are allowed) with vertex set $X\left(\vec{G}\right)=\left\{x_{1},x_{2},\ldots ,x_{n}\right\}$ and edge set $E(\vec{G})$. For every vertex *x*_*i*_ ∈ *X* we associate two numbers: the *in-degree* and the *out-degree* of *x*_*i*_. These numbers form the directed degree bi-sequence **D**.

We transform the directed graph $\vec{G}$ into the following *bipartite representation*: let $B\left(\vec{G}\right)=\left(U,V;E\right)$ be a bipartite graph where each class consists of one copy of every vertex from $X(\vec{G})$. The edges adjacent to a vertex *u*_*x*_ in class *U* represent the out-edges from *x*, while the edges adjacent to a vertex *v*_*x*_ in class *V* represent the in-edges to *x* (so a directed edge *xy* corresponds the edge *u*_*x*_*v*_*y*_). If a vertex has zero in- (respectively out-) degree in $\vec{G}$, then we delete the corresponding vertex from $B(\vec{G})$. (Actually, this representation is an old trick used by Gale [5], but one can find it already in [2]). The directed degree bi-sequence **D** gives rise to a bipartite degree sequence.

Here we make good use of the notion of *chords*: since there are no loops in our directed graph, there cannot be any (*u*_*x*_, *v*_*x*_) edge in its bipartite representation—these vertex pairs are **non-chords**. It is easy to see that these forbidden edges form a forbidden (partial) matching F in the bipartite graph $B(\vec{G})$, or in more general terms, in *B*(**D**). To make it easier to remember the nature of restriction, we will denote this **restricted** bipartite degree sequence with $\vec{d}$.

We consider all realizations $G(\vec{d})$ which avoid the non-chords from F. Now it is easy to see that the bipartite graphs in $G(\vec{d})$ are in one-to-one correspondence with the possible realizations of the directed degree bi-sequence.

Consider now again our example about two oppositely oriented triangles, $\vec{C_{3}}$ and $\overset{\leftarrow }{C_{3}}$. Consider the bipartite representations $B(\vec{C_{3}})$ and $B(\overset{\leftarrow }{C_{3}})$, and take their symmetric difference ∇. It contains exactly one alternating cycle (the edges come alternately from $B(\vec{C_{3}})$ and $B(\overset{\leftarrow }{C_{3}})$), s.t. each vertex pair of distance 3 along the cycle in ∇ forms a non-chord. Therefore, in this alternating cycle a “classical” swap cannot be performed. To address this issue, we introduce a new swap operation: we exchange all edges coming from $B(\vec{C_{3}})$ with all edges coming from $B(\overset{\leftarrow }{C_{3}})$ in one operation. The corresponding operation for directed graphs is exactly the triple swap operation.

In general: if the current symmetric difference ∇ contains a length-6 alternating cycle *C*_6_ such that all opposite vertex pairs form non-chords, then we allow performing the corresponding *C*_6_-**swap**. In this notation, the original swap should properly be called a *C*_4_-**swap** (for obvious reasons), but for the sake of simplicity we only write swap instead of *C*_4_-swap. By the constraints posed by the forbidden partial matching, only a subset of all bipartite swaps can be performed. These swaps together with the possible *C*_6_-swaps we just defined are called the F-**compatible** swaps or F-**swaps** for short.

**Lemma 6** ([18], [12]). *The set* $G\left(B\left(D\right)\right)=G\left(\vec{d}\right)$ *of all realizations is irreducible under* F-*swaps*.

We are ready to define our swap Markov chain $\vec{M}=\left(G\left(\vec{d}\right),P\right)$ for the restricted bipartite degree sequence $\vec{d}$.

The transition (probability) matrix *P* of the Markov chain is defined as follows: let the current realization be *G*. Then

1. with probability 1/2 we stay in the current state, so our Markov chain is lazy;
2. with probability 1/4 we uniformly choose two-two vertices *u*_1_, *u*_2_;*v*_1_, *v*_2_ from classes *U* and *V* respectively and perform the swap if it is possible;
3. finally, with probability 1/4 we choose three-three vertices from *U* and *V* and check whether they form three pairs of forbidden chords. If this is the case, then we perform a *C*_6_-swap if it is possible.

The swap moving from *G* to *G*′ is unique, therefore the probability of this transformation (the *jumping probability* from *G* to *G*′ ≠ *G*) is:

Prob(G→bG′):=P(G′|G)=14·1(|U|2)(|V|2), (9)

and

Prob(G→cG′):=P(G′|G)=14·1(|U|3)(|V|3). (10)

(These probabilities reflect the fact that *G*′ should be derived from *G* by a *C*_4_-swap or by a *C*_6_-swap). The probability of transforming *G* to *G*′ (or vice versa) is time-independent and symmetric. Therefore, *P* is a symmetric matrix, where the entries in the main diagonal are non-zero, but (possibly) distinct values. Our Markov chain is irreducible (by Lemma 6), and it is clearly aperiodic, since it is lazy. Therefore, as it is well-known, the Markov process $\vec{M}$ is reversible with the uniform distribution as the globally stable stationary distribution.

## The general properties of the swap Markov chain on bipartite degree sequences

The proofs of our theorems closely follow the proof of Theorem 10 in [12], which, in turn, is based on the proof method developed in [10]. Suppose $\vec{d}$ is a directed degree sequence and **d** is the degree sequence of bipartite representations corresponding to the realizations of $\vec{d}$. As we saw earlier the sets of all realizations G(d) and $G(\vec{d})$ are slightly different: while $G\left(\vec{d}\right)\subset G\left(d\right)$ but there are realizations in G(d) that contain edges which are forbidden in the realizations in $G(\vec{d}).$ However, the following reasoning from [12] applies to both bipartite and directed degree sequences. Therefore the notation G is used to refer either of the two realization sets.

Consider two realizations X,Y∈G, and take the symmetric difference ∇ = *E*(*X*)Δ*E*(*Y*). Now for each vertex in the bipartite graph (*U*, *V*; ∇) the number of incident *X*-edges (= *E*(*X*)\*E*(*Y*)) and the number of the incident *Y*-edges are equal. Therefore ∇ can be decomposed into alternating circuits and later into alternating cycles. The way the decomposition is performed is described in detail in Section 5 of the paper [10]. Here we just summarize the highlights:

First, we decompose the symmetric difference ∇ into alternating circuits in all possible ways. In each case we get an ordered sequence *W*_1_, *W*_2_, …, *W*_*κ*_ of circuits. Each circuit is endorsed with a fixed cyclic order.

Now we fix one circuit decomposition. Each circuit *W*_*i*_ in the ordered decomposition has a unique alternating cycle decomposition: $W_{i}=C_{1}^{i},C_{2}^{i},\ldots ,C_{k_{i}}^{i}$. (This unique decomposition is a quite delicate point and was discussed in detail in Section 5.2 of the paper [10]).

The ordered circuit decomposition of ∇ together with the ordered cycle decompositions of all circuits provide a well-defined ordered cycle decomposition *C*_1_, …, *C*_*l*_ of ∇. This decomposition does not depend on any swap operations, only on the symmetric difference of realizations *X* and *Y*.

This ordered cycle decomposition singles out *l* − 1 different realizations *H*_1_, …, *H*_*l*−1_ from G with the following property: for each *j* = 0, …, *l* − 1 we have *E*(*H*_*j*_)Δ*E*(*H*_*j*+1_) = *C*_*j*+1_ if we apply the notation *H*_0_ = *X* and *H*_*l*_ = *Y*. This means that

$$\begin{array}{c} E\left(H_{i}\right)=E\left(X\right)△\left(\underset{i^{\prime }\leq i}{\cup }E\left(C_{i^{\prime }}\right)\right). \end{array}$$

It remains to design a unique canonical path from *X* to *Y* determined by the circuit decomposition, which uses the realizations *H*_*j*_ as **milestones** along the path. In other words, for each pair *H*_*j*_, *H*_*j*+1_ we should design a swap sequence which turns *H*_*j*_ into *H*_*j*+1_.

Here we slightly abuse the general naming conventions: in the original canonical path method for any pair X,Y∈G exactly one *X* → *Y* path is defined. In Sinclair’s multicommodity flow method ([19]) a (usually large) set of paths is defined, equipped with a probability distribution. In our presentation we use the expression *canonical path* to denote these paths, differentiating the paths in G and the paths in some realizations.

So, the canonical path under construction is a sequence

$$\begin{array}{c} X=G_{0},\ldots ,G_{i},\ldots ,G_{m}=Y \end{array}$$

of realizations, where each *G*_*i*_ can be derived from *G*_*i*−1_ with exactly one swap operation, and there exists an increasing subscript subsequence 0 = *n*_0_ < *n*_1_ < *n*_2_ < ⋯ < *n*_*ℓ*_ = *m*, such that we have $G_{n_{k}}=H_{k}$ for every 0 ≤ *k* ≤ *ℓ*.

## The construction of swap sequences between consecutive milestones

Next we define the canonical path corresponding to the cycle *C*_*i*_. The procedure described here is slightly different from the one in [12], since the excluded edge set F in [12] is slightly larger than the one used here.

For convenience, we will use the names $G,G^{\prime }\in G$ instead of *H*_*i*_ and *H*_*i*+1_. These two graphs have almost the same edge set:

$$\begin{array}{cc} (E\left(G\right)\backslash (C_{i}\cap E\left(X\right)))\cup (C_{i}\cap E\left(Y\right))= & E\left(G^{\prime }\right)\\ (E\left(G^{\prime }\right)\backslash \left(C_{i}\cap E\left(Y\right)\right))\cup (C_{i}\cap E\left(X\right))= & E\left(G\right). \end{array}$$

We refer to the elements of *C*_*i*_ ∩ *E*(*X*) as *X*-edges, while the rest of the edges of *C*_*i*_ are *Y*-edges. We denote the cycle *C*_*i*_ by C, which has 2*ℓ* edges and its vertices are *u*_1_, *v*_1_, *u*_2_, *v*_2_, …, *u*_*ℓ*_, *v*_*ℓ*_. Finally, w.l.o.g. we may assume that the chord *u*_1_*v*_1_ is a *Y*-edge (and, of course, *v*_*ℓ*_*u*_1_ is an *X*-edge).

We will build our canonical path from *G* towards *G*′. At any particular step, the last constructed realization is denoted by *Z*. (At the beginning of the process we have *Z* = *G*). We are looking for the next realization, denoted by *Z*′. We will control the canonical path system with an **auxiliary** structure, originally introduced by Kannan, Tetali and Vempala in [1]:

The matrix *M*_*G*_ denotes the **adjacency matrix** of the bipartite realization *G* where the rows and columns are indexed by the vertices of *U* and *V*, respectively, with the slight alteration that a position corresponding to a forbidden edge (a non-chord) is indicated with a *. There is a natural correspondence between the entries of matrices on *U* × *V* and the chords of *G*. Our auxiliary structure is the matrix

$$\begin{array}{c} \hat{M}\left(X+Y-Z\right)=M_{X}+M_{Y}-M_{Z}. \end{array}$$

Summation does not change the positions with a *. Since the non-* entries of a bipartite adjacency matrix are 0 or 1, the possible entries of $\hat{M}$ are *, −1, 0, 1, 2. An entry is * if it corresponds to a forbidden edge, and it is −1 if the edge is missing from both *X* and *Y* but it exists in *Z*. It is 2 if the edge is missing from *Z* but exists in both *X* and *Y*. It is 1 if the edge exists in all three graphs (*X*, *Y*, *Z*) or it is there only in one of *X* and *Y* but not in *Z*. Finally, it is 0 if the edge is missing from all three graphs, or the edge exists in exactly one of *X* and *Y* and in *Z*. (Therefore, if an edge exists in exactly one of *X* and *Y* then the corresponding chord in $\hat{M}$ is always 0 or 1). It is easy to see that the row and column sums of $\hat{M}\left(X+Y-Z\right)$ are the same as the row and column sums in *M*_*X*_ (or *M*_*Y*_, or *M*_*Z*_).

Now we are ready to determine the F-swap sequence between *G* and *G*′ and this is the point where realizations from G(d) and $G(\vec{d})$ start behave slightly differently. From now on we will work with realizations from $G(\vec{d})$ but we will point out those turning points where there are real differences. The first such difference is that in the case of a directed realization $G\in G(\vec{d})$ there may be a vertex *v*_*i*_ along the cycle *C* s.t. *u*_1_*v*_*i*_ is a non-chord, while for a simple bipartite realization G∈G(d) this does not happen.

**Fig 1:**
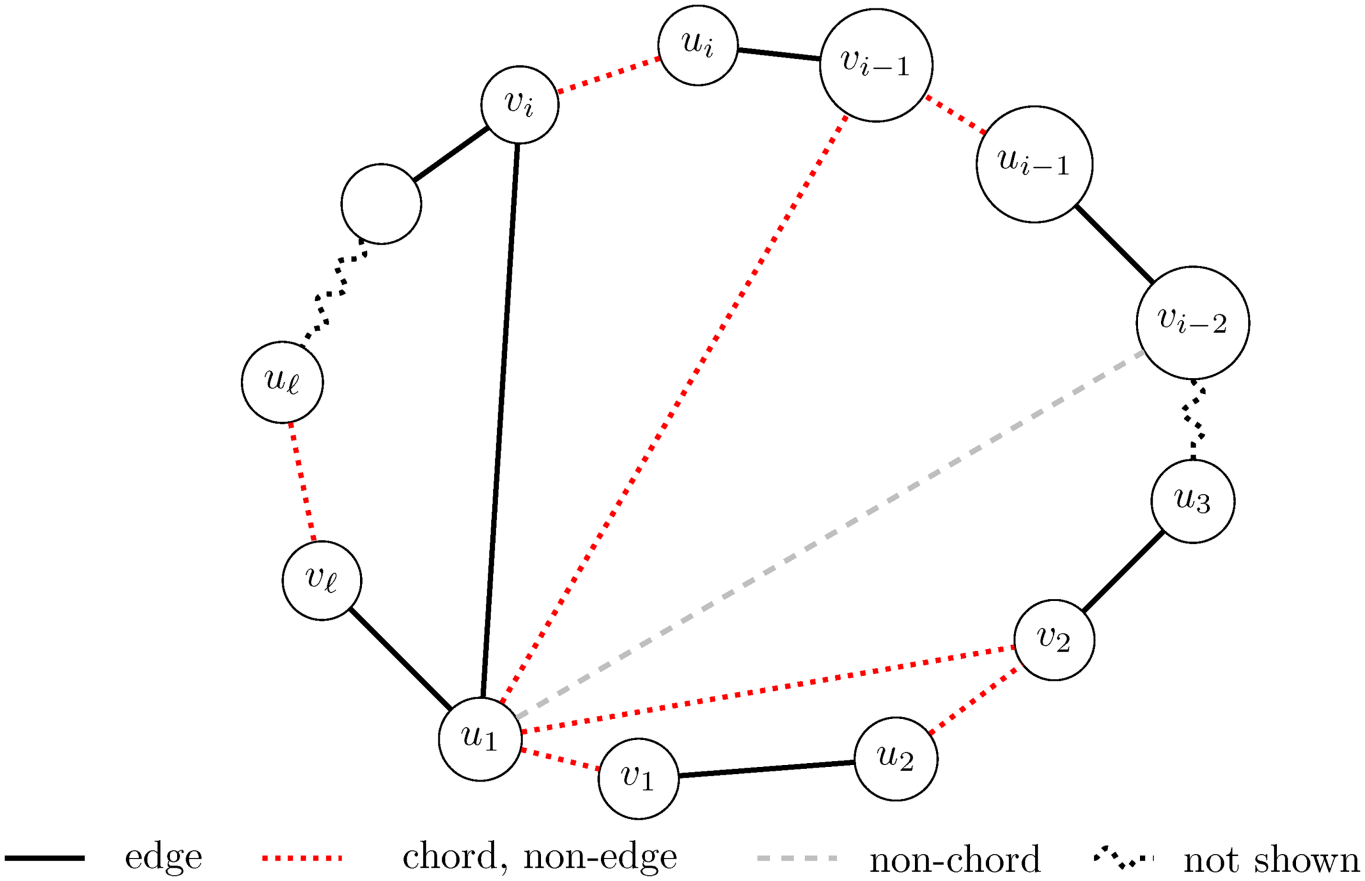
Sweeping a cycle.

We determine the F-swap sequence between *G* and *G*′ from $G(\vec{d})$ through an iterative algorithm. In the first iteration we check, step by step, the positions (*u*_1_, *v*_2_), (*u*_1_, *v*_3_), …, (*u*_1_, *v*_*ℓ*_) and take the smallest *j* for which (*u*_1_, *v*_*i*_) is an actual edge in *G*. Since (*u*_1_, *v*_*ℓ*_) is an edge in *G*, such an *i* always exists. A typical configuration is shown in Fig 1.

We call the chord *u*_1_*v*_*i*_ the **start-chord** of the current sub-process and *u*_1_*v*_1_ is the **end-chord**. We will **sweep** the alternating chords along the cycle. The vertex *u*_1_ will be the **cornerstone** of this operation. This process works from the start-chord *u*_1_*v*_*i*_, *v*_*i*_*u*_*i*_ (non-edge), *u*_*i*_*v*_*i*−1_ (an edge) toward the end-chord *v*_1_*u*_1_ (non-edge)—switching their status in twos and fours. We check positions *u*_1_*v*_*i*−1_, *u*_1_*v*_*i*−2_ (all are non-edges) and choose the first chord among them, which we call the **current-chord**. (Since *u*_1_ belongs to at most one non-chord we never have to check more than two positions to find a chord).

**Case 1**: As we just explained the typical situation is that the current-chord is the “next” one, so when we start this is typically *u*_1_*v*_*i*−1_. Assume that this is a chord. Then we can proceed with the swap operation *v*_*i*−1_*u*_*i*_, *v*_*i*_*u*_1_ ⇒ *u*_1_*v*_*i*−1_, *u*_*i*_*v*_*i*_. We just produced the first “new” realization in our sequence, this is $G_{1}^{\prime }$. For the next swap operation this will be our new current realization. This operation will be called a **single-step**.

In a realization *Z* we call a chord **bad** if its state in *Z* (being edge or non-edge) is different from its state in *G*, or equivalently, different from its state in *G*′, since *G* and *G*′ differ only on the chords along the cycle C (recall that in our nomenclature a chord is a pair of vertices which may form an edge). After the previous swap, we have two bad chords in $G_{1}^{\prime }$, namely *u*_1_*v*_*i*−1_ and *v*_*i*_*u*_1_.

Consider now the auxiliary matrix $\hat{M}\left(X+Y-Z\right)$ (here $Z=G_{1}^{\prime }$). As we saw earlier, any chord not contained in C has the same state in *X*, *Y* and *Z*. Accordingly, the corresponding matrix value is 0 or 1 in $\hat{M}$. We call a position **bad** in $\hat{M}$ if this value is −1 or 2. (A bad position in $\hat{M}$ always corresponds to a bad chord). Since we switch the start-chord into a non-edge, it may become 2 in $\hat{M}$ (in case the start-chord is an edge in both *X* and *Y*). Furthermore, the current-chord turned into an edge. If it is a non-edge in both *X* and *Y* then its corresponding value in $\hat{M}$ becomes −1. After this step, we have at most two bad positions in the matrix, at most one with 2-value and at most one with −1-value. Finishing our swap operation, the previous current-chord becomes the new start-chord, so it is the edge *u*_1_, *v*_*i*−1_.

**Case 2**: If the position below the start-chord (this is now *u*_1_*v*_*i*−2_) is a non-chord, then we cannot produce the previous swap. Then the non-edge *u*_1_*v*_*i*−3_ is the current-chord. For sake of simplicity we assume that *i* − 3 = 2 so we are in Fig 1. (That is, *i* − 1 = 4). Consider now the alternating *C*_6_ cycle: *u*_1_, *v*_2_, *u*_3_, *v*_3_, *u*_4_, *v*_4_. It has altogether three vertex pairs which may be used to perform an F-swap operation. We know already that *u*_1_*v*_3_ is a non-chord. If neither *v*_2_*u*_4_ nor *u*_3_*v*_4_ are chords, then this alternating cycle provides an F-compatible circular *C*_6_-swap. Again, we found the valid swap *v*_2_*u*_3_, *v*_3_*u*_4_, *v*_4_*u*_1_ ⇒ *u*_1_*v*_2_, *u*_3_*v*_3_, *u*_4_*v*_4_. After that we again have 2 bad chords, namely *u*_1_*v*_2_ and *v*_4_*u*_1_, and together we have at most two bad positions in the new $\hat{M}\left(X+Y-Z\right)$, with at most one 2-value and at most one −1-value.

Finally, if one position, say *v*_2_*u*_4_, is a chord then we can process this *C*_6_ with two swap operations. If this chord is, say, an actual edge, then we swap *v*_2_*u*_4_, *v*_4_*u*_1_ ⇒ *u*_1_*v*_2_, *u*_4_*v*_4_. After this we can take care of the *v*_2_, *u*_3_, *v*_3_, *u*_4_ cycle. Along this sequence we never create more than 3 bad chords: the first swap makes chords *v*_2_*u*_4_, *v*_4_*u*_1_, and *u*_1_*v*_2_ bad ones, and the second cures *v*_2_*u*_4_ but does not touch *u*_1_*v*_2_ and *v*_4_*u*_1_. So, along this swap sequence we have 3 bad chords, and in the end we have only 2. On the other hand, if the chord *v*_2_*u*_4_ is not an edge, then we can swap *v*_2_*u*_3_, *v*_3_*u*_4_ ⇒ *u*_3_*v*_3_, *u*_4_*v*_2_, creating one bad edge, then by swapping the four cycle *u*_1_, *v*_2_, *u*_4_, *v*_4_ we cure *v*_2_*u*_4_ but we switch *u*_1_*v*_2_ and *v*_4_*u*_1_ into bad chords. We finished our **double-step** along the cycle.

In a double-step we create at most three bad chords. When the first swap uses three chords along the cycle then we may have at most one bad chord (with $\hat{M}$-value 0 or −1) and then the next swap switches back the chord into its original status, and makes two new bad chords (with at most one 2-value and one −1-value). When the first swap uses only one chord from the cycle, then it creates three bad chords (changing two chords into non-edges and one into an edge), therefore it may create at most two 2-values and one −1-value. After the second swap, there will be only two bad chords, with at most one 2-value, and at most one −1-value.

When only the third position corresponds to a chord in our *C*_6_ then after the first swap we may have two −1-values and one 2-value. However, after the next swap we will have at most one of both types.

After finishing our single- or double-step, the previous current-chord becomes the new start-chord and we look for the new current-chord. Then we repeat our procedure. There is one important point to be mentioned: along the step, the start-chord switches back into its original status, so it will not be a bad chord anymore. So even if we face a double-step the number of bad chords will never be larger than three (together with the chord *v*_*i*_*u*_1_ which is still in the wrong state, so it is a bad chord), and we always have at most two 2-values and at most one −1-value in $\hat{M}\left(X+Y-Z\right)$.

When our current-chord becomes *v*_1_*u*_2_ then the last step will switch back the last start-chord into its correct state, and the last current-chord cannot be in a bad state. So, when we finish our sweep from *u*_1_*v*_*i*_ to *v*_1_*u*_1_, we will only have one bad chord (with a possible 2-value in $\hat{M}$). This concludes the first iteration of our algorithm.

For the next iteration, we seek a new start-chord between *v*_*i*_*u*_1_ and *v*_*ℓ*_*u*_1_ and the chord *v*_*i*_*u*_1_ becomes the new end-chord. We repeat our sweeping procedure until there are no more unprocessed chords. Upon completion, we find a realization sequence from *G* to *G*′. If in the first sweep we had a double-step, then such a step will never occur later, so altogether with the (new) bad end-chord we never have more than three bad chords (corresponding to at most two 2-values and at most one −1-value).

However, if the double-step occurs sometime later, for example in the second sweep, then we face to the following situation: if we perform a circular *C*_6_-swap, then all together we have at most two 2-values and one −1-value. Thus, we may assume that there is a chord suitable for a swap in our *C*_6_. If this chord is a non-edge, then the swap around it produces one bad chord, and at most one bad position in $\hat{M}$. The only remaining case is when that chord is an edge. After the first swap there will be four bad chords, and there may be at most three 2-values and at most one −1 value. However, after the next swap (finishing the double step) we annihilate one of the 2-values, and after that swap there are at most two 2-values and at most one −1-value along the entire swap sequence. When we finish our second sweep, then chord *v*_*i*_*u*_1_ will be switched back into its original state and it will not be bad anymore.

Iteratively applying the algorithm, the entire cycle C is processed after at most *ℓ* sweep sequences. This finishes the construction of the required F-swap sequence (and the required realization sequence).

Meanwhile we also proved the following important observations:

**Lemma 7**. *For the Markov chain* M, *we always have at most two* 2-*values and at most one* −1-*value in our auxiliary matrix* $\hat{M}\left(X+Y-Z\right)$ *along our procedure*.

**Lemma 8**. *For the Markov chain* $\vec{M}$, *each auxiliary matrix* $\hat{M}\left(X+Y-Z\right)$ *occurring along our procedure is at most swap-distance one from a matrix with at most three bad positions: with at most two* 2-*values and with at most one* −1-*value in the same column*.

Now we are ready to describe the following, highly technical theorem from [10] which is required to show that the defined swap Markov-chains are rapidly mixing.

**Theorem 9** (Section 4 in [10]). *If the designed canonical path system satisfies the three conditions below, then the MCMC process is rapidly mixing. The conditions are*:

(Θ) *For each i* < *l the constructed path* $H_{i}=G_{0}^{\prime },G_{1}^{\prime },\ldots ,G_{m^{\prime }}^{\prime }=H_{i+1}$ *satisfies m*′ ≤ *c* ⋅ |*C*_*i*+1_| *for a suitable constant c*.
(Ω) ∀_*j*_ *there exists a realization* $K_{j}\in V\left(G\right)$ s.t. $d(M_{X}+M_{Y}-M_{G_{j}^{\prime }},M_{K_{j}})$ ≤ Ω_2_, *where M_G_ is the **bipartite adjacency matrix** of G*, d *denotes the Hamming distance, and* Ω_2_ *is a small constant*.
(Ξ) *For each vertex* $G_{j}^{\prime }$ *in the path being traversed the following three objects together uniquely determine the realizations X, Y and the path itself*:
  - *The auxiliary matrix* $M_{X}+M_{Y}-M_{G_{j}^{\prime }}$,
  - *the symmetric difference* ∇ = *E*(*X*)△*E*(*Y*),
  - *and a polynomial size parameter set* B.

The meaning of condition (Ξ) is that these structures can be used to control certain features of the canonical path system; namely, their numbers give a bound on the number of canonical paths between any realization pair *X*, *Y* which traverses $G_{j}^{\prime }$.

Condition (Ω) implies that the space of auxiliary matrices is larger than V(G) by a multiplicative factor of at most ${\left(nm\right)}^{2\Omega_{2}}$.

To use this theorem we have to show that the defined swap sequences between *H*_*i*_ and *H*_*i*+1_, using the cornerstone *u*_1_ chosen in (Φ), satisfy conditions (Θ), (Ω), and (Ξ) of Theorem 9. The first one is easy to see, since we can process any cycle of length 2*ℓ* in *ℓ* − 1 swaps. Therefore, we may choose *c* = 1 in (Θ). Condition (Ξ) holds for the same reason as it holds in paper [12]. Thus only condition (Ω) remains to be checked.

Until this very moment the choice of the cornerstone vertex *u*_1_ was arbitrary. Before we turn to the analysis of the swap sequences, we choose which particular vertex of the cycle C will serve as its cornerstone.

Let the submatrix *A* contain those positions from any adjacency or any auxiliary matrix which correspond to the positions *u*_*i*_*v*_*j*_ defined by the vertices from C. Furthermore, denote by *A*[*Z*] the submatrix of $\hat{M}\left(X+Y-Z\right)$ spanned by the vertices of C. Then:

(Φ) Let *u*_1_ be a vertex which has the lowest row sum in the submatrix *A*[*H*_*i*_] = *A*[*G*].

## The analysis of the swap sequences between milestones in M

In this section, we will analyze the undirected case. We introduce the new **switch** operation on integer matrices: we fix the four corners of a submatrix, and we add 1 to two corners in a diagonal, and add −1 to the corners on the other diagonal. This operation clearly does not change the column and row sums of the matrix. (We will use this operation on adjacency matrices or on auxiliary matrices of realizations). For example, if we consider the adjacency matrix *M*_*G*_ of a realization of **d** and make a valid swap operation, then this is equivalent to a switch in this matrix. The next statement is trivial but very useful:

**Claim 10**. *If two matrices have **switch-distance** 1, then their Hamming distance is* 4. *Consequently, if the switch-distance is c then the Hamming distance is bounded by* 4*c*.

The next lemma shows that property (Ω) holds for the auxiliary matrices along the swap sequence from *G* toward *G*′ for degree sequences corresponding to Theorem 3 and Theorem 2.

**Lemma 11**. *For any realization Z along the constructed swap sequence from G to G*′ *in* G(d) *there exists a realization K* = *K*(*Z*) *such that*

$$\begin{array}{c} d(\hat{M}\left(X+Y-Z\right),M_{K})\leq 16. \end{array}$$

*Proof*. The swap sequence transforming *G* to *G*′ only touches chords induced by V(C). Therefore, the row and column sums in *A*[*Z*] are the same as that of *A*[*G*], so the cornerstone has the minimum row sum in *A*[*Z*] as well.

Any entries of 2’s and −1’s in $\hat{M}$ are in the row of *u*_1_, moreover, they are contained in *A*[*Z*]. Suppose ${\hat{M}}_{u_{1},v_{j}}=2$. The sum of entries of *A*[*Z*] in the column *v*_*j*_ is <|U∩V(C)|=|V∩V(C)|, therefore $\exists \;u_{k}\in U\cap V\left(C\right)$ such that ${\hat{M}}_{u_{k},v_{j}}=0$. Since the sum of the entries in row *u*_1_ is minimum among the rows of *A*[*Z*], there must $\exists v_{l}\in V\cap V\left(C\right)$ such that ${\hat{M}}_{u_{k},v_{l}}>{\hat{M}}_{u_{1},v_{l}}$. Obviously, ${\hat{M}}_{u_{k},v_{l}}<2$, so ${\hat{M}}_{u_{1},v_{l}}\in \left\{0,-1\right\}$. The switch operation *u*_1_*v*_*j*_, *u*_*k*_*v*_*l*_ ⇒ *u*_1_*v*_*l*_, *u*_*k*_*v*_*j*_ (decrease the entries of the matrix by one at positions *u*_1_*v*_*j*_ and *u*_*k*_*v*_*l*_, and increase the entries at positions *u*_1_*v*_*l*_ and *u*_*k*_*v*_*j*_ by one) in $\hat{M}$ (and in *A*[*Z*]) eliminates the entry of 2 at *u*_1_*v*_*j*_, and creates an entry of 1 at both *u*_1_*v*_*j*_ and *u*_*k*_*v*_*j*_. In the column *v*_*l*_ three scenarios are possible: either the entry −1 and a 0 exchange their positions, or a 0 and a 1 exchange their positions; finally, it is also possible that the −1 and a 1 both become 0.

By repeating the previous argument, we may eliminate one more entry 2, if necessary, from *A*[*Z*]. (Recall that at the beginning we had at most two 2s in $\hat{M}$). Although it is possible that the entry −1 is not in the *u*_1_-row anymore, it does not cause any hardship. Let ${\hat{M}}^{\prime }$ be the matrix we get after performing these at most two switches that eliminate the 2’s. Each entry of ${\hat{M}}^{\prime }$ is a 0 or a 1, except at most one −1 entry.

The proof now diverges into two cases corresponding to Theorem 2 and Theorem 3, respectively.

### Case of Theorem 2: Sequence d satisfies Eq 1

Suppose that ${\hat{M}}_{u_{0},v_{0}}^{\prime }=-1$. Since both *u*_0_ and *v*_0_ are at least 1, there ∃*v*_1_ ∈ *V* and ∃*u*_1_ ∈ *U* such that ${\hat{M}}_{u_{0},v_{1}}^{\prime }={\hat{M}}_{u_{1},v_{0}}^{\prime }=1$. If ${\hat{M}}_{u_{1},v_{1}}^{\prime }=0$, there is a switch which transforms ${\hat{M}}^{\prime }$ into a realization of **d**. Otherwise, observe that

$$\begin{array}{c} |\{\left(u,v\right)|u\in U\backslash \left\{u_{0},u_{1}\right\},v\in V\backslash \left\{v_{0},v_{1}\right\},{\hat{M}}_{u,v}^{\prime }={\hat{M}}_{u_{1},v}^{\prime }={\hat{M}}_{u,v_{1}}^{\prime }=1\}|\leq \\ \leq \left(d\left(v_{1}\right)-2\right)\cdot \left(\Delta -1\right)+\left(d\left(u_{1}\right)-2\right)\cdot \left(\Delta -1\right)\leq \\ \leq 2(\Delta -1)(\Delta -2). \end{array}$$

The number of entries of 1 in ${\hat{M}}^{\prime }$ that are incident on the same row as *u*_0_ or *u*_1_, on the same column as *v*_0_ or *v*_1_, or in the above counted set is at most

$$\begin{array}{c} 2\left(\Delta +1\right)+2\left(\Delta -1\right)-1+2\left(\Delta -1\right)\left(\Delta -2\right)\leq 2\Delta^{2}-2\Delta +3<\left|E\right|. \end{array}$$

The last inequality follows from Equality 1. Therefore there exists *u*_2_ ∈ *U*, *v*_2_ ∈ *V* such that {*u*_0_, *v*_0_, *u*_1_, *v*_1_, *u*_2_, *v*_2_} is a set of 6 vertices where ${\hat{M}}_{u_{2},v_{2}}^{\prime }=1$ and ${\hat{M}}_{u_{2},v_{1}}^{\prime }={\hat{M}}_{u_{1},v_{2}}^{\prime }=0$. Switching along the six positions determined by the cyclically successive pairs, we get an adjacency matrix corresponding to a realization of **d**.

### Case of Theorem 3: Sequence d satisfies Eqs 2 and 3

From now on we will consider the entire matrix ${\hat{M}}^{\prime }$ and not only *A*. Suppose that ${\hat{M}}_{u_{0},v_{0}}^{\prime }=-1$. Let $U^{\prime }=\left\{u\in U\;|\;{\hat{M}}_{u,v_{0}}^{\prime }=1\right\}$ and $V^{\prime }=\left\{v\in V\;|\;{\hat{M}}_{u_{0},v}^{\prime }=1\right\}$. If ∃(*u*, *v*) ∈ *U*′ × *V*′ such that ${\hat{M}}_{u,v}^{\prime }=0$, then switch operation *u*_0_*v*, *uv*_0_ ⇒ *u*_0_*v*_0_, *uv* transforms ${\hat{M}}^{\prime }$ into an adjacency matrix.

Suppose from now on, that ∀(*u*, *v*)∈*U*′ × *V*′ we have ${\hat{M}}_{u,v}^{\prime }=1$. Let

$$\begin{array}{cc} U^{\prime \prime } & =\{u\in U\;|\;\exists v\in V^{\prime }\;:\;{\hat{M}}_{u,v}^{\prime }=0\},\\ V^{\prime \prime } & =\{v\in V\;|\;\exists u\in U^{\prime }\;:\;{\hat{M}}_{u,v}^{\prime }=0\}. \end{array}$$

Clearly, *U*″ ∩ *U*′ = *V*″ ∩ *V*′ = ∅. Suppose there ∃(*u*_2_, *v*_2_) ∈ *U*″ × *V*″ such that ${\hat{M}}_{u_{2},v_{2}}^{\prime }=1$. By definition, there ∃(*u*_1_, *v*_1_) ∈ *U*′ × *V*′ such that ${\hat{M}}_{u_{2},v_{1}}^{\prime }=0$ and ${\hat{M}}_{u_{1},v_{2}}^{\prime }=0$. Clearly, applying first the switch operation *u*_1_, *u*_2_ and *v*_1_, *v*_2_, and then the operation *u*_0_, *u*_1_ and *v*_0_, *v*_1_ transforms ${\hat{M}}^{\prime }$ into an adjacency matrix.

**Fig 2:**
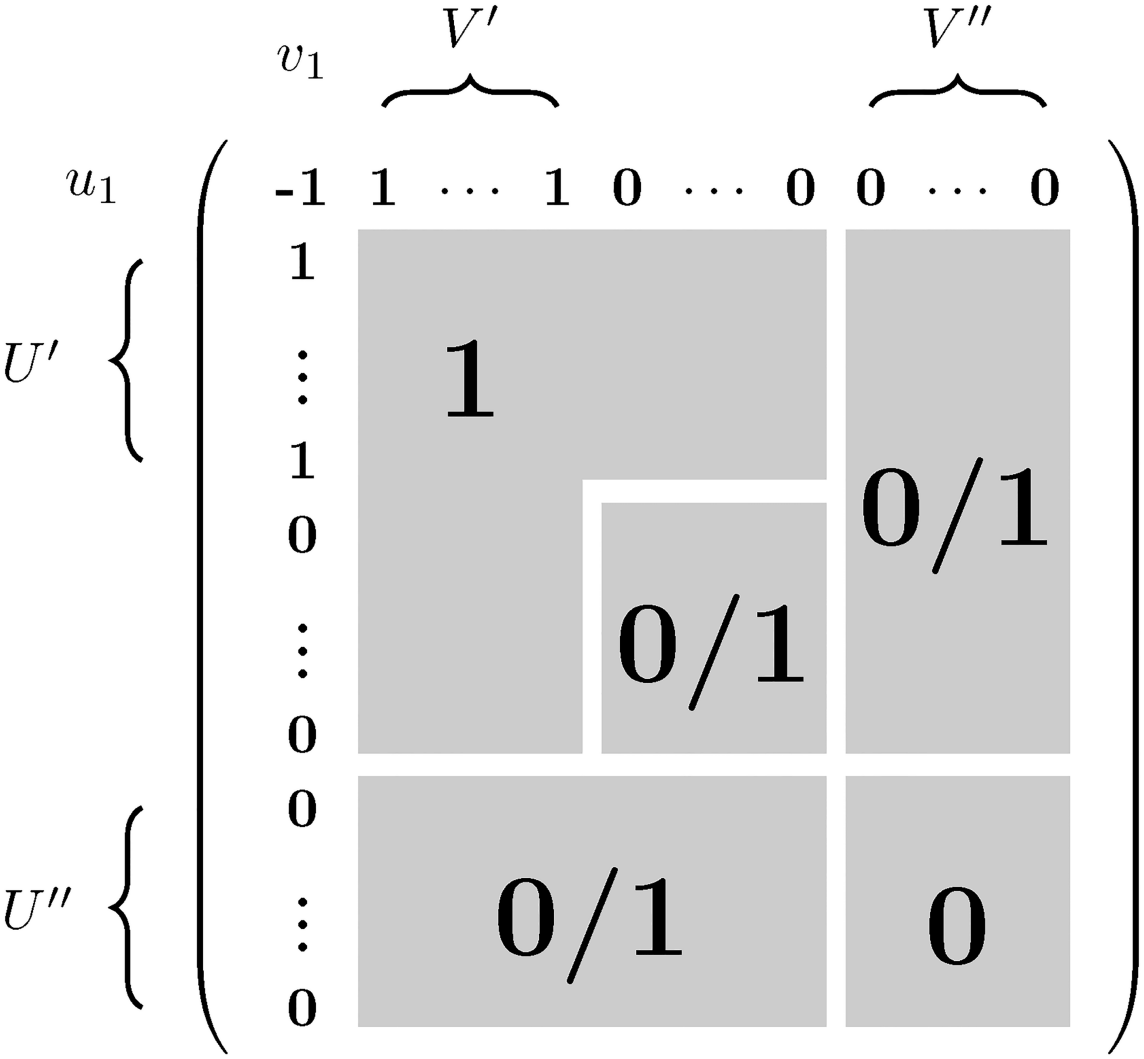
${\hat{M}}^{\prime }$ is shown; each of the entries in the regions marked with 0/1 may be 0 or 1.

Lastly, suppose that ∀(*u*, *v*)∈*U*″ × *V*″ we have ${\hat{M}}_{u,v}^{\prime }=0$. This case is shown in Fig 2.

In addition to the zeroes in *U*″ × *V*″, ${\hat{M}}_{u,v_{0}}^{\prime }=0$ for any *u* ∈ *U*″. We have

$$\begin{array}{c} \begin{array}{cc} |U^{\prime \prime }| & \cdot \left(m-d_{1}\right)\geq |\{\left(u,v\right)\in U^{\prime \prime }\times V\;|\;{\hat{M}}_{u,v}^{\prime }=0\}|=\\ & =|U^{\prime \prime }\times V^{\prime \prime }\left|+\right|U^{\prime \prime }|+|\{\left(u,v\right)\in U^{\prime \prime }\times \left(V\backslash V^{\prime \prime }\backslash \left\{v_{0}\right\}\right)|\;{\hat{M}}_{u,v}^{\prime }=0\}|. \end{array} \end{array}$$ (11)

The right-hand side can be estimated from below as follows. Since the row and column sums of ${\hat{M}}^{\prime }$ are the same as that of *M*_*X*_, we have

$$\begin{array}{c} |U^{\prime }|\geq c_{1}-{\hat{M}}_{u_{0},v_{0}}^{\prime }=c_{1}+1,\;\text{and}\;\left|V^{\prime }\right|\geq d_{1}-{\hat{M}}_{u_{0},v_{0}}^{\prime }=d_{1}+1. \end{array}$$

For any *v* ∈ *V*′ and *u* ∈ *U*\*U*″, we have ${\hat{M}}_{u,v}^{\prime }=1$. Also, for any *u* ∈ *U*′ and *v* ∈ *V*\*V*″, we have ${\hat{M}}_{u,v}^{\prime }=1$. Therefore

$$\begin{array}{cc} n-c_{1}-2\geq \left|U\backslash U^{\prime }\backslash \left\{u_{0}\right\}\right|\geq & |U^{\prime \prime }|\geq n-c_{2},\\ m-d_{1}-2\geq \left|V\backslash V^{\prime }\backslash \left\{u_{0}\right\}\right|\geq & |V^{\prime \prime }|\geq m-d_{2}. \end{array}$$

Clearly, if *c*_2_ ≤ *c*_1_ + 1 or *d*_2_ ≤ *d*_1_ + 1 (i.e., *G* is almost half-regular), we already have a contradiction. We also have

$$\begin{array}{c} \begin{array}{cc} & |\{\left(u,v\right)\in U^{\prime \prime }\times \left(V\backslash V^{\prime \prime }\backslash \left\{v_{0}\right\}\right)|\;{\hat{M}}_{u,v}^{\prime }=0\}|\geq \\ & \geq \left(n-c_{2}\right)\left(m-\right|V^{\prime \prime }\left|-1\right)-|U\backslash U^{\prime }\backslash U^{\prime \prime }|\cdot |V\backslash V^{\prime }\backslash V^{\prime \prime }\backslash \left\{v_{0}\right\}|\geq \\ & \geq \left(n-c_{2}\right)\left(m-\right|V^{\prime \prime }\left|-1\right)-(n-c_{1}-1-\left|U^{\prime \prime }\right|)\cdot (m-d_{1}-2-\left|V^{\prime \prime }\right|). \end{array} \end{array}$$ (12)

Combining Eqs 11 and 12,

$$\begin{array}{c} |U^{\prime \prime }|\cdot \left(m-d_{1}\right)\geq \left(n-c_{2}\right)\cdot (m-|V^{\prime \prime }\left|-1)+\right|U^{\prime \prime }|\left(m-d_{1}-1\right)+\\ \;\;+\left(\right|V^{\prime \prime }\left|+1\right)\cdot \left(n-c_{1}-1\right)-\left(n-c_{1}-1\right)\cdot \left(m-d_{1}-1\right). \end{array}$$

Further simplifying:

$$|U^{\prime \prime }|\geq \left(\right|V^{\prime \prime }\left|+1\right)\left(c_{2}-c_{1}-1\right)+\left(c_{1}+1-c_{2}\right)m+\left(d_{1}+1\right)n-\left(c_{1}+1\right)\left(d_{1}+1\right).$$

Since we may suppose that *c*_2_ ≥ *c*_1_ + 2, we can substitute |*V*″| ≥ *m* − *d*_2_ and |*U*″| ≤ *n* − *c*_1_ − 2 into the inequality, yielding

$$\left(c_{2}-c_{1}-1\right)\left(d_{2}-d_{1}-1\right)\geq d_{1}\left(n-c_{2}\right)+1.$$

Symmetrically, a similar derivation gives

$$\left(c_{2}-c_{1}-1\right)\left(d_{2}-d_{1}-1\right)\geq c_{1}\left(n-d_{2}\right)+1.$$

The last two inequalities clearly contradict the assumptions of this claim.

In summary, in every case there exist at most 4 switches which transform $\hat{M}$ into a 0 − 1 matrix, which is a matrix with suitable row- and column sums, therefore it is the adjacency matrix of a realization *K* of the degree sequence **d**.

## The analysis of the swap sequences between milestones in $\vec{M}$

Now we turn to discussing the directed case. As in the previous section, condition (Ω) is the only remaining assumption of Theorem 9 which does not immediately follow from the construction of swap sequences between consecutive milestones.

**Lemma 12**. *For any realization Z along the constructed swap sequence from G to G*′ *there exists a realization K* = *K*(*Z*) *such that*

$$\begin{array}{c} d(\hat{M}\left(X+Y-Z\right),M_{K})\leq 20. \end{array}$$

*Proof*. As described by Lemma 8, it is possible that realization *Z* is derived by an F-swap which is a first *C*_4_-swap to resolve an alternating *C*_6_ cycle along the sweep. It may introduce an extra 2-value and/or a −1-value into the auxiliary structure. But Lemma 8 also shows that the next *C*_4_ swap will revert these extra bad positions. Therefore let *Z*^*S*^ denote the realization *Z* itself if this extra swap is not needed, or the new realization if it is needed. Then $\hat{M}\left(X+Y-Z^{S}\right)$ has at most two entries of 2 and at most one entry of −1. Now we have to show that there is a realization *K* such that

$$\begin{array}{c} d(\hat{M}\left(X+Y-Z^{S}\right),M_{K})\leq 16. \end{array}$$

As before we will use the shorthand $\hat{M}\left(X+Y-Z^{S}\right)=\hat{M}.$

The swap sequence transforming the bipartite representation *G* to *G*′ (also, the previous extra swap) only touches chords induced by V(C). Therefore, the row and column sums in *A*[*Z*] are the same as that of *A*[*G*], so the cornerstone has the minimum row sum in *A*[*Z*] as well.

Any entries of 2’s and −1’s in $\hat{M}$ are in the row of *u*_1_, moreover, they are contained in *A*[*Z*]. Suppose ${\hat{M}}_{u_{1},v_{j}}=2$. The column of *v*_*j*_ in *A*[*Z*] contains at least one zero, therefore there exist two vertices $u_{k},u_{k^{\prime }}\in U\cap V\left(C\right)$ such that ${\hat{M}}_{u_{k},v_{j}}=0$ and ${\hat{M}}_{u_{k^{\prime }},v_{j}}=0$, even if there is a * in the column of *v*_*j*_. We have two cases.

**Fig 3:**
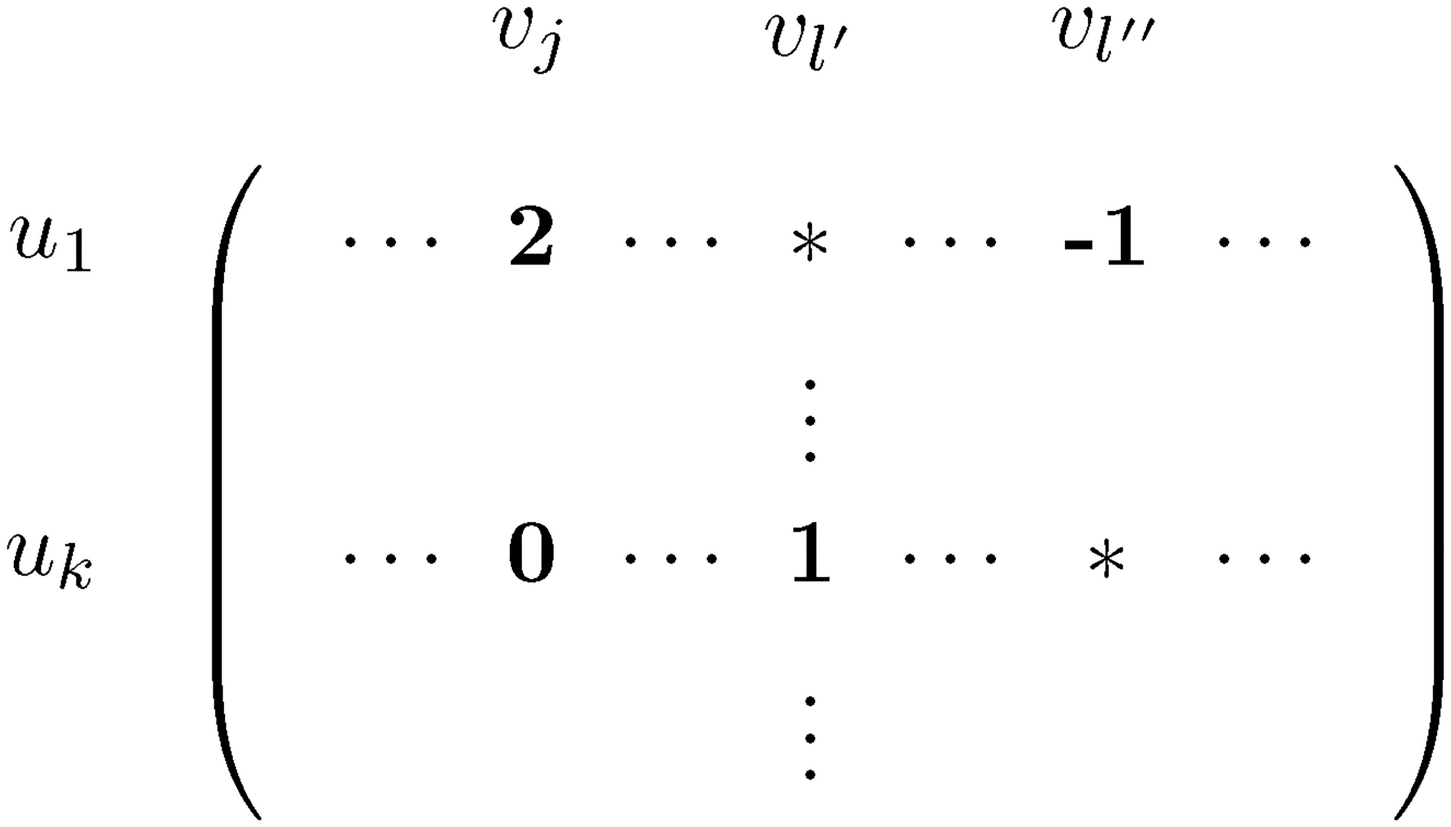
$\hat{M}$ is shown for Case 2 of the proof of Lemma 12.

1. There $\exists v_{l}\in V\cap V\left(C\right)$ such that ${\hat{M}}_{u_{k},v_{l}}>{\hat{M}}_{u_{1},v_{l}}$: obviously, ${\hat{M}}_{u_{k},v_{l}}<2$, so ${\hat{M}}_{u_{1},v_{l}}\in \left\{0,-1\right\}$. The switch operation *u*_1_*v*_*j*_, *u*_*k*_*v*_*l*_ ⇒ *u*_1_*v*_*l*_, *u*_*k*_*v*_*j*_ (decrease the entries of the matrix by one at positions *u*_1_*v*_*j*_ and *u*_*k*_*v*_*l*_, and increase the entries at positions *u*_1_*v*_*l*_ and *u*_*k*_*v*_*j*_ by one) in $\hat{M}$ (and in *A*[*Z*]) eliminates the entry of 2 at *u*_1_*v*_*j*_, and creates an entry of 1 at both *u*_1_*v*_*j*_ and *u*_*k*_*v*_*j*_. In the column *v*_*l*_ three scenarios are possible: either the entry −1 and a 0 exchange their positions, or a 0 and a 1 exchange their positions; finally, it is also possible that the −1 and a 1 both become 0.
2. If for all $v_{l}\in V\cap V\left(C\right)$ either ${\hat{M}}_{u_{k},v_{l}}\leq {\hat{M}}_{u_{1},v_{l}}$ or ${\hat{M}}_{u_{k},v_{l}}=*$ or ${\hat{M}}_{u_{1},v_{l}}=*$ holds: since the sum of the entries in row *u*_1_ is minimum among the rows of *A*[*Z*], this is only possible if there exist *v*_*l*′_, *v*_*l*″_ ∈ *V* ∩ *V*(*C*) such that ${\hat{M}}_{u_{1},v_{l^{\prime }}}=*$, ${\hat{M}}_{u_{k},v_{l^{\prime }}}=1$, ${\hat{M}}_{u_{1},v_{l^{\prime \prime }}}=-1$, ${\hat{M}}_{u_{k},v_{l^{\prime \prime }}}=*$, and for $v_{l}\in V\cap V\left(C\right)\backslash \left\{v_{j},v_{l^{\prime }},v_{l^{\prime \prime }}\right\}$ we have ${\hat{M}}_{u_{k},v_{l}}={\hat{M}}_{u_{1},v_{l}}$. This is shown on Fig 3.

If the second case applies, the first case must hold if we replace *k* by *k*′; if not, column *v*_*l*″_ would contain two *, a contradiction.

By repeating the previous argument, we may eliminate one more entry 2, if necessary, from *A*[*Z*] (and $\hat{M}$). (Recall that at the beginning we had at most two 2’s in $\hat{M}$). Although it is possible that the entry −1 is not in the *u*_1_-row anymore, it does not cause any hardship. Let ${\hat{M}}^{\prime }$ be the matrix we get after performing these at most two switches that eliminate the 2’s. Each entry of ${\hat{M}}^{\prime }$ is a 0 or a 1, except at most one −1 entry.

The proof now diverges into two cases corresponding to Theorem 4 and Theorem 5, respectively.

### Case of Theorem 4: Sequence d satisfies Eq 4

From now on we will consider the entire matrix ${\hat{M}}^{\prime }$ and not only *A*. Suppose that ${\hat{M}}_{u_{0},v_{0}}^{\prime }=-1$. The degrees of *u*_0_ and *v*_0_ are at least one, and so there are at least two entries of 1’s in the row and column of *u*_0_ and *v*_0_, respectively. Therefore there exists *u*_1_ ∈ *U* and *v*_1_ ∈ *V* such that they are not copies of the same original vertex and ${\hat{M}}_{u_{0},v_{1}}^{\prime }={\hat{M}}_{u_{1},v_{0}}^{\prime }=1$. If ${\hat{M}}_{u_{1},v_{1}}^{\prime }=0$, there is a switch which transforms ${\hat{M}}^{\prime }$ into a bipartite realization of $\vec{d}$. Otherwise ${\hat{M}}_{u_{1},v_{1}}^{\prime }=1$, so observe that

$$\begin{array}{c} |\{\left(u,v\right)|u\in U\backslash \left\{u_{0},u_{1}\right\},v\in V\backslash \left\{v_{0},v_{1}\right\},{\hat{M}}_{u,v}^{\prime }=1,({\hat{M}}_{u_{1},v}^{\prime }=0\;\text{or}\;{\hat{M}}_{u,v_{1}}^{\prime }=0)\}|\leq \\ \leq \left(d_{in}\left(v_{1}\right)-2\right)\cdot \left(\Delta -1\right)+\left(d_{out}\left(u_{1}\right)-2\right)\cdot \left(\Delta -1\right)\leq \\ \leq 2(\Delta -1)(\Delta -2). \end{array}$$

The number of entries of 1 in ${\hat{M}}^{\prime }$ that are incident on either the rows of *u*_0_ or *u*_1_, on the column of *v*_0_ or *v*_1_, or on a position corresponding to an element of the above counted set is at most

$$\begin{array}{c} 2\left(\Delta +1\right)+2\left(\Delta -1\right)+2\left(\Delta -1\right)\left(\Delta -2\right)\leq 2\Delta^{2}-2\Delta +4. \end{array}$$

This implies that there exist *u*_2_ ∈ *U* and *v*_2_ ∈ *V* such that ${\hat{M}}_{u_{2},v_{2}}^{\prime }=1$, ${\hat{M}}_{u_{1},v_{2}}^{\prime }\neq 1$, and ${\hat{M}}_{u_{2},v_{1}}^{\prime }\neq 1$. Because Equality 4 claims $|\vec{E}|$ is strictly larger than the right hand side by at least 2Δ, we may also assume that ${\hat{M}}_{u_{1},v_{2}}^{\prime }\neq *$, and ${\hat{M}}_{u_{2},v_{1}}^{\prime }\neq *$. Therefore there exists *u*_2_ ∈ *U*, *v*_2_ ∈ *V* such that {*u*_0_, *v*_0_, *u*_1_, *v*_1_, *u*_2_, *v*_2_} is a set of 6 vertices where ${\hat{M}}_{u_{2},v_{2}}^{\prime }=1$ and ${\hat{M}}_{u_{2},v_{1}}^{\prime }={\hat{M}}_{u_{1},v_{2}}^{\prime }=0$. Switching along the six positions determined by the cyclically successive pairs, we get an adjacency matrix corresponding to a realization of **d**.

### Case of Theorem 5: Sequence d satisfies Eqs 5 and 6

From now on we will consider the entire matrix ${\hat{M}}^{\prime }$ and not only *A*. Suppose that ${\hat{M}}_{u_{0},v_{0}}^{\prime }=-1$. Let $U^{\prime }=\left\{u\in U\;|\;{\hat{M}}_{u,v_{0}}^{\prime }=1\right\}$ and $V^{\prime }=\left\{v\in V\;|\;{\hat{M}}_{u_{0},v}^{\prime }=1\right\}$. If ∃(*u*, *v*) ∈ *U*′ × *V*′ such that ${\hat{M}}_{u,v}^{\prime }=0$, then switch operation *u*_0_*v*, *uv*_0_ ⇒ *u*_0_*v*_0_, *uv* transforms ${\hat{M}}^{\prime }$ into an adjacency matrix.

Suppose from now on, that ∀(*u*, *v*)∈*U*′ × *V*′ we have ${\hat{M}}_{u,v}^{\prime }\in \left\{1,*\right\}$. Let

$$\begin{array}{cc} U^{\prime \prime } & =\{u\in U\;|\;\exists v\in V^{\prime }\;:\;{\hat{M}}_{u,v}^{\prime }=0\},\\ V^{\prime \prime } & =\{v\in V\;|\;\exists u\in U^{\prime }\;:\;{\hat{M}}_{u,v}^{\prime }=0\}. \end{array}$$

Clearly, *U*″ ∩ *U*′ = *V*″ ∩ *V*′ = ∅. Suppose ${\hat{M}}^{\prime }\left[U^{\prime \prime },V^{\prime \prime }\right]$ contains more than |*U*″| entries of 1’s. A simple pigeon-hole principle argument implies that there ∃(*u*_2_, *v*_2_) ∈ *U*″ × *V*″ and ∃(*u*_1_, *v*_1_) ∈ *U*′ × *V*′ such that ${\hat{M}}_{u_{2},v_{2}}^{\prime }=1$, ${\hat{M}}_{u_{2},v_{1}}^{\prime }=0$, ${\hat{M}}_{u_{1},v_{2}}^{\prime }=0$, and $M_{u_{1},v_{1}}^{\prime }=1$. Clearly, applying first the switch operation *u*_1_, *u*_2_ and *v*_1_, *v*_2_, and then the operation *u*_0_, *u*_1_ and *v*_0_, *v*_1_ transforms ${\hat{M}}^{\prime }$ into an adjacency matrix.

Lastly, suppose that ${\hat{M}}^{\prime }\left[U^{\prime \prime },V^{\prime \prime }\right]$ contains at most |*U*″| entries of 1’s. We have

$$\begin{array}{c} |U^{\prime \prime }|\cdot \left(n-d_{1}\right)-\left|U^{\prime \prime }\right|\geq \\ \geq |\{\left(u,v\right)\in U^{\prime \prime }\times V\;|\;{\hat{M}}_{u,v}^{\prime }=0\}|\geq \\ \geq (|U^{\prime \prime }\times V^{\prime \prime }\left|-\right|U^{\prime \prime }|)+|\{\left(u,v\right)\in U^{\prime \prime }\times \left(V\backslash V^{\prime \prime }\right)|\;{\hat{M}}_{u,v}^{\prime }\in \left\{0,*\right\}\}|-\left|U^{\prime \prime }\right|, \end{array}$$

since ${\hat{M}}^{\prime }\left[U^{\prime \prime },V\right]$ contains exactly |*U*″| of *. The right-hand side can be estimated from below as follows. Since the row and column sums of ${\hat{M}}^{\prime }$ are the same as that of *M*_*X*_, we have

$$\begin{array}{c} |U^{\prime }|\geq c_{1}-{\hat{M}}_{u_{0},v_{0}}^{\prime }=c_{1}+1,\;\text{and}\;\left|V^{\prime }\right|\geq d_{1}-{\hat{M}}_{u_{0},v_{0}}^{\prime }=d_{1}+1. \end{array}$$

Also,

$$\begin{array}{c} n-c_{1}-2\geq \left|U\backslash U^{\prime }\backslash \left\{u_{0}\right\}\right|\geq |U^{\prime \prime }|\geq n-c_{2}-1,\\ n-d_{1}-2\geq \left|V\backslash V^{\prime }\backslash \left\{u_{0}\right\}\right|\geq |V^{\prime \prime }|\geq n-d_{2}-1. \end{array}$$

Clearly, if *c*_2_ = *c*_1_ or *d*_2_ = *d*_1_ (i.e., *G* is half-regular), we already have a contradiction. Each column in *V* \ *V*″ may contain at most one *, therefore

$$\begin{array}{c} |\{\left(u,v\right)\in U^{\prime \prime }\times \left(V\backslash V^{\prime \prime }\backslash \left\{v_{0}\right\}\right)|\;{\hat{M}}_{u,v}^{\prime }\in \left\{0,*\right\}\}|\geq \\ \;\;\geq \left(n-c_{2}\right)\left(n-\right|V^{\prime \prime }\left|-1\right)-|U\backslash U^{\prime }\backslash U^{\prime \prime }|\cdot |V\backslash V^{\prime }\backslash V^{\prime \prime }\backslash \left\{v_{0}\right\}|. \end{array}$$

Moreover, ${\hat{M}}_{u,v_{0}}^{\prime }\in \left\{0,*\right\}$ for any *u* ∈ *U*″. Combining these inequalities, we get

$$\begin{array}{c} |U^{\prime \prime }|\cdot \left(n-d_{1}\right)\geq (|U^{\prime \prime }\times V^{\prime \prime }\left|-\right|V^{\prime \prime }|)+\left|U^{\prime \prime }\right|+\\ +\left(n-c_{2}\right)\left(n-\right|V^{\prime \prime }\left|-1\right)-|U\backslash U^{\prime }\backslash U^{\prime \prime }|\cdot |V\backslash V^{\prime }\backslash V^{\prime \prime }\backslash \left\{v_{0}\right\}|. \end{array}$$

A few lines of computation similar to those in the previous section give

$$\begin{array}{c} \left(c_{2}-c_{1}\right)\left(d_{2}-d_{1}\right)\geq d_{1}\left(n-c_{2}-1\right)+3+c_{1}+d_{2}-n. \end{array}$$

Symmetrically, we also have

$$\begin{array}{c} \left(c_{2}-c_{1}\right)\left(d_{2}-d_{1}\right)\geq c_{1}\left(n-d_{2}-1\right)+3+d_{1}+c_{2}-n. \end{array}$$

The last two inequalities clearly contradict the assumptions of this claim.

In summary, in every case there exist at most 4 switches which transform $\hat{M}$ into a 0 − 1 matrix, which is a matrix with suitable row- and column sums, therefore it is the adjacency matrix of a realization *K* of the degree sequence $\vec{d}$.

## Erdős-Rényi random graphs

The following statement is a straightforward, easy consequence of Theorem 3.

**Corollary 13**. *If G is a bipartite Erdős-Rényi random graph on vertex classes of size n and m, with edge probability p*(*n*, *m*), *such that*

$$\begin{array}{c} 3\cdot \sqrt{\frac{\log m+\frac{1}{2}\log2}{n}}\leq p\left(n,m\right)\leq 1-3\cdot \sqrt{\frac{\log n+\frac{1}{2}\log2}{m}}, \end{array}$$

*then the swap Markov chain is rapidly mixing on the bipartite degree sequence of G with probability at least* $1-\frac{1}{n}-\frac{1}{m}$. *(The roles of m and n can be interchanged)*.

*Proof*. Let *p* = *p*(*n*, *m*), $\varepsilon_{c}=\frac{1}{3}pn$ and $\varepsilon_{d}=\frac{1}{3}\left(1-p\right)m$. Also, let *c*_1_ = *pn* − *ε*_*c*_, *c*_2_ = *pn*+ *ε*_*c*_, *d*_1_ = *pm* − *ε*_*d*_, *d*_2_ = *pm*+ *ε*_*d*_. Eq 3 holds, we only need to check that

$$4\varepsilon_{c}\varepsilon_{d}\leq (pn-\varepsilon_{c})\cdot (m-pm-\varepsilon_{d}).$$

Moreover, by Hoeffding’s inequality,

$$\begin{array}{c} \mathrm{Pr}(\text{Equation}\;2\;\text{does}\;\text{not}\;\text{hold})\leq \\ \leq \mathrm{Pr}(\exists v\in V\;|d\left(v\right)-pn|>\varepsilon_{c})+\mathrm{Pr}(\exists u\in U\;|d\left(u\right)-pm|>\varepsilon_{d})\leq \\ \leq m\cdot 2e^{-2p^{2}n/9}+n\cdot 2e^{-2{\left(1-p\right)}^{2}m/9}\leq \frac{1}{m}+\frac{1}{n}, \end{array}$$

which proves the statement.

For completeness sake, we also state the respective theorem for directed random graphs.

**Corollary 14**. *If* $\vec{D}$ *is a directed Erdős-Rényi random graph on n vertices with out-edge probability p*(*n*), *such that*

$$\begin{array}{c} 3\cdot \sqrt{\frac{\log n+\frac{1}{2}\log2}{n}}+\frac{2}{\sqrt{n}}\leq p\left(n\right)\leq 1-3\cdot \sqrt{\frac{\log n+\frac{1}{2}\log2}{n}}-\frac{2}{\sqrt{n}}, \end{array}$$

*then the swap Markov chain is rapidly mixing on the directed degree sequence of* $\vec{D}$ *with probability at least* $1-\frac{2}{n}$.

## Comparing the applicability of the Theorems

Next we discuss the applicability of our results. Theorem 2 (and the result of Greenhill and Sfragara) is not applicable, when the average degree $\bar{d}>\frac{n}{2}$. In case of Theorem 3 there is no such region: for example if all degrees are between $\frac{n}{3}+1$ and $\frac{2n}{3}-1$, then inequality $\bar{d}>\frac{n}{2}$ for the average degree is possible. It is also easy to see that Theorem 3 applies to all half-regular (consequently for all regular) bipartite degree sequences.

However, if the degrees are evenly distributed between 1 and $\frac{n}{4}$, then Theorem 2 applies while Theorem 3 does not. Therefore these results have different validity regions and they are independent from each other.

Generally speaking Theorem 2 and the Greenhill and Sfragara results are better applicable to degree sequences developed under some scale-free random dynamics (with *γ* > 2.5), while Theorem 3 is better fitted to degree sequences developed under the Erdős—Rényi model.

For directed degree sequences similar analysis applies.
